# Evaluating the impact of COVID-19 on cancer care: a comprehensive analysis of treatment modifications, risk factors, and patient outcomes

**DOI:** 10.1186/s12879-025-11172-2

**Published:** 2025-06-02

**Authors:** Xuquan Jing, Min Wang, Shuangqing Lu, Jiling Niu, Feihu Chen, Hanjing Yin, Haoyu Liu, Dongmei Sun, Hui Zhu

**Affiliations:** Department of Radiation Oncology, Shandong Cancer Hospital and Institute, Shandong First Medical University and Shandong Academy of Medical Sciences, Jinan, 250117 Shandong Province China

**Keywords:** COVID-19, Cancer, Chemotherapy, Radiotherapy, Immunotherapy, Tyrosine kinase inhibitors, Anti-vascular therapy

## Abstract

**Background:**

Cancer patients will have an increased incidence of corona virus disease 2019 (COVID-19) infection. The severity of COVID-19 infection varies among cancer patients who have other complications and are being treated.

**Method:**

This retrospective cohort study evaluated the impact of cancer treatments on COVID-19 incidence and outcomes in 603 cancer patients. Key objectives included assessing treatment-related risks, severity, and treatment efficacy, along with the effects of treatment interruptions on patient recovery and therapy resumption.

**Result:**

This study included 603 cancer patients, of whom 68 (11.28%) were infected with COVID-19, and 398 (66%) were vaccinated. Logistic regression analysis revealed that underlying comorbidities, chemotherapy, and radiotherapy were significantly associated with an increased risk of COVID-19 infection (*P* < 0.05 for chemotherapy and radiotherapy). Factors such as sex, smoking status, cancer pathology, and staging showed no significant correlation with COVID-19 incidence. Treatment disruptions during the pandemic were observed, with chemotherapy being most affected (42.86% suspension), while surgery and TKI therapy showed minimal interruption. Efficacy analysis indicated no significant difference in treatment outcomes between suspended and uninterrupted treatments (*P* = 0.758). Treatment suspensions did not significantly alter toxicity profiles, with bone marrow suppression being the most frequent toxicity.

**Conclusion:**

Chemotherapy and radiotherapy increased the risk of COVID-19 in cancer patients, with treatment interruptions not affecting efficacy or toxicity, underscoring the need for tailored management.

## Introduction

Coronavirus disease 2019 (COVID-19), caused by the novel severe acute respiratory syndrome coronavirus 2 (SARS-CoV-2), has become a global public health crisis [1]. As a newly emerged infectious disease, COVID-19 poses a significant threat to populations without prior immunity, particularly to individuals who are elderly or have underlying conditions such as diabetes, coronary heart disease, and hypertension [2, 3]. Cancer patients are at increased risk of both COVID-19 infection and mortality, with clinical studies demonstrating a significantly higher infection rate and case fatality rate compared to the general population [4]. Specifically, a case fatality rate of 5.6% was reported in cancer patients, nearly three times higher than the overall population’s rate [5, 6].

Epidemiological data reveal that cancer patients represent approximately 2% of all individuals infected with SARS-CoV-2, with the majority being elderly, predominantly in the 76 to 85-year age group (36.5%), and male (59.5%). Among these patients, solid tumors account for 59% of cases, and 54% are in an active stage of their cancer [7]. The tumor disease itself, coupled with ongoing cancer treatments such as chemotherapy and radiotherapy, further elevates the risk of severe outcomes in COVID-19-infected patients. Periodic treatments like chemoradiotherapy may compromise immune function, potentially enhancing vulnerability to infections such as COVID-19 [7]. A study found that cancer patients infected with COVID-19 had a significantly higher mortality rate (41.3% vs. 17.2%, *P* = 0.0001) compared to non-cancer patients [8]. The risk of death was notably increased in cancer patients, independent of other factors. Cancer patients also exhibited fewer common symptoms such as fever, chills, and cough. Another study demonstrated that cancer patients infected with COVID-19 had a significantly higher rate of mortality and mechanical ventilation compared to patients without a cancer history [9]. Specifically, 41.7% of cancer patients died from COVID-19, compared to 6.8% in the non-cancer group, and 25.0% of cancer patients required mechanical ventilation, compared to 4.7% in the control group.

Despite these observations, there is a lack of definitive clinical data directly supporting this notion.

Studies have shown that the main clinical manifestations of COVID-19 are fever, cough, and dyspnea [10–12]. While the clinical manifestations of COVID-19 in cancer patients are generally similar to those in non-cancer patients, cancer patients are more likely to experience dyspnea and exhibit higher levels of pro-inflammatory cytokines, such as TNF-α, IL-6, and IL-2R, as well as significant decreases in lymphocytes and T cells [12, 13]. Moreover, the clinical presentations of COVID-19 pneumonia and immune-associated pneumonia are often similar, with both conditions showing symmetrical patchy ground-glass opacities (GGO) and consolidation areas on CT scans. To distinguish between these conditions, routine laboratory tests and CT imaging are recommended after RT-PCR confirmation [13, 14]. In addition, elevated C-reactive protein (CRP) and erythrocyte sedimentation rate (ESR) are commonly observed in patients with immune-associated pneumonia, while D-dimer, liver enzymes, lactate dehydrogenase (LDH), myoglobin, and troponin levels are increased in severe cases [13, 15].

In conclusion, while previous research has highlighted various risk factors associated with COVID-19 infection—such as disease type, critical illness indicators, long-term complications, and the risk of reinfection—there remains ongoing debate regarding the impact of prior cancer treatments on the incidence and severity of COVID-19 in cancer patients. This study seeks to address this gap by analyzing data from clinical questionnaires collected from cancer patients who underwent chemotherapy and radiotherapy during the pandemic. The results provide compelling evidence of a significant association between these cancer treatments and an increased susceptibility to COVID-19 infection.

## Methods

### Study design and objectives

This retrospective cohort study aimed to evaluate the relationship between cancer treatment regimens and the incidence, severity, and outcomes of COVID-19 infection in cancer patients. The primary objectives were to assess the impact of chemotherapy, radiotherapy, targeted therapy, immunotherapy, antiangiogenic therapy, and other treatments on the likelihood of contracting COVID-19, and to examine the outcomes of infection, including disease severity, treatment efficacy, and toxicity. The secondary objectives included evaluating changes in treatment outcomes following interruptions due to the COVID-19 pandemic and identifying the impact of these treatments on patient recovery, treatment suspension rates, and resumption of therapy.

### Study population

The study included 603 cancer patients diagnosed with SARS-CoV-2 infection at Shandong Cancer Hospital and Institute between December 2019 and 2023. COVID-19 diagnosis was confirmed using either nucleic acid testing (RT-PCR) or chest CT scans, with the timing of infection and the onset of symptoms, including fever, cough, and sore throat, recorded. The inclusion criteria required that patients be diagnosed with cancer and have a history of at least one cancer treatment, including chemotherapy, radiotherapy, targeted therapy, immunotherapy, or anti-angiogenesis therapy. Patients who had not received any anti-cancer treatment during the study period or were undergoing surgery were excluded. Demographic information, clinical characteristics, and cancer-specific data (including cancer type, stage, and Karnofsky Performance Status (KPS)) were collected for each patient.

### COVID-19 diagnosis and clinical management

Detailed data on the diagnosis and clinical management of COVID-19 were collected for all enrolled patients. COVID-19 diagnosis was confirmed by nucleic acid testing (RT-PCR) or chest CT scans, with the timing of infection and the onset of symptoms, such as fever, cough, and sore throat, documented. Patients diagnosed with COVID-19-associated myocarditis underwent further evaluations, including hematological assessments (e.g., BNP, TNT), blood coagulation profiles, and electrocardiogram abnormalities. Information on COVID-19 treatments, including antiviral agents, corticosteroids, and supportive care, was also recorded, along with any interruptions in cancer treatment due to COVID-19. Furthermore, vaccination status was documented, noting whether patients had been vaccinated and the type of vaccine administered.

### Cancer treatment regimens

Detailed information was gathered on the types of anti-tumor treatments received by the patients. These treatments included chemotherapy (67.50%), radiotherapy (32.01%), immunotherapy (40.80%), targeted therapy (17.58%), and anti-angiogenic therapy (18.74%). Patients were grouped based on whether they had received these treatments, and the relationship between treatment modality and the risk of contracting COVID-19, as well as disease severity, was analyzed. Additionally, the timing of treatment relative to COVID-19 infection was recorded to assess any impact on infection rates. Patients who underwent surgery were excluded from the analysis to focus on systemic treatments.

### Endpoints

The primary endpoint was the incidence of COVID-19 infection among cancer patients, categorized by treatment modality. Secondary endpoints included the severity of COVID-19 infection, defined by progression to severe pneumonia, need for intensive care, and mortality. Additional endpoints were the efficacy of anti-tumor treatments following the infection, including Complete Response (CR), Partial Response (PR), Stable Disease (SD), and Progressive Disease (PD). The analysis also assessed treatment toxicity profiles, including fatigue, electrolyte disturbances, dermatitis, secondary pneumonia, hepatic or renal impairment, gastrointestinal reactions, and bone marrow suppression, particularly in relation to treatment interruptions.

### Data collection

Data were collected using customized data collection forms that included information on demographics, comorbidities, cancer type, treatment regimens, and the timing of treatment interruptions. The occurrence of COVID-19 infections was documented, as well as any related interruptions in anti-tumor therapies. The data was independently reviewed by two investigators to ensure accuracy and completeness. This study was approved by the Ethics Committee of Shandong Cancer Hospital and Institute.

### Data statistics

Statistical analysis was performed using SPSS 26.0. Descriptive statistics were used to summarize demographic characteristics, with continuous data expressed as medians and quartile ranges, and categorical data presented as frequencies and percentages. Univariate analysis was performed to assess the association between various demographic factors (such as age, gender, smoking status, and comorbidities) and the incidence of COVID-19 infection. Chi-square (χ^2^) test were used to evaluate the association between treatment modalities and COVID-19 incidence.

Multivariate logistic regression analysis was conducted to identify independent risk factors for the occurrence of COVID-19 infection and its severity. Variables included in the multivariate model were age, gender, comorbid conditions, cancer type, cancer stage, and KPS, as these have been identified as potential confounders in the relationship between cancer treatment and COVID-19 outcomes. A *P*-value of < 0.05 was considered statistically significant.

## Results

### Basic data

#### Basic patient information

This study analyzed 603 cancer patients, of whom 68 (11.28%) were infected with COVID-19, while 535 (88.72%) were not (Table 1). The patient population was predominantly male (67.50%), with females comprising 32.50%. Most patients were under 65 years old (64.34%), and 35.66% were 65 or older. A significant proportion of patients had a Karnofsky Performance Status (KPS) score above 80 (97.51%).

**Table 1:** Basic patient information

Name	Category	Counts	Percentage (%)	Cumulative Percentage (%)
Sex	male	407	67.50	67.50
	female	196	32.50	100.00
Age	< 65 yr	388	64.34	64.34
	≥ 65 yr	215	35.66	100.00
KPS score	0<80	15	2.49	2.49
	≥ 80	588	97.51	100.00
Smoke	No	352	58.37	58.37
	Yes	251	41.63	100.00
Hypertension	No	471	78.11	78.11
	Yes	132	21.89	100.00
Myocardial infarction	No	588	97.51	97.51
	Yes	15	2.49	100.00
Cerebral infarction	No	590	97.84	97.84
	Yes	13	2.16	100.00
Kidney injury	No	602	99.83	99.83
	Yes	1	0.17	100.00
Diabetes	No	552	91.54	91.54
	Yes	51	8.46	100.00
Coronary heart disease	No	581	96.35	96.35
	Yes	22	3.65	100.00
Operation History	No	591	98.01	98.01
	Yes	12	1.99	100.00
Disease of respiratory system	No	583	96.68	96.68
	Yes	20	3.32	100.00
Others	No	584	96.85	96.85
	Yes	19	3.15	100.00
Basic Disease	without	391	64.84	64.84
	1	156	25.87	90.71
	≥ 2	56	9.29	100.00
Pathology	Adenocarcinoma	329	54.56	54.56
	Squamous carcinoma	107	17.74	72.31
	Small cell carcinoma	139	23.05	95.36
	Other carcinoma	28	4.64	100.00
Staging	0	5	0.83	0.83
	1	21	3.48	4.31
	2	20	3.32	7.63
	3	171	28.36	35.99
	4	386	64.01	100.00
Ttotal		603	100.0	100.0

Regarding comorbidities, 58.37% of patients were non-smokers, and 41.63% were smokers. Hypertension was present in 21.89% of patients, while 78.11% were free of it. Diabetes affected 8.46%, and other conditions such as myocardial infarction, cerebral infarction, and kidney injury were rare (2.49%, 2.16%, and 0.17%, respectively). Most patients had no underlying diseases (64.84%), while 25.87% had one, and 9.29% had two or more. The most common cancer type was adenocarcinoma (54.56%), followed by small cell carcinoma (23.05%), squamous cell carcinoma (17.74%), and others (4.64%). Interestingly, patients with more advanced cancer, particularly stage 4 (64.01%), were more susceptible to COVID-19, highlighting the potential link between cancer progression and increased vulnerability to infections.

#### Treatment patterns

Among the cohort, 48.26% had received two prior treatments, 36.48% one, and 15.26% three or more. Surgical interventions were rare (2.82%, Table 2). Chemotherapy was the most common prior treatment (67.50%), while 32.50% had not received it. Radiotherapy and immunotherapy were less frequent, administered to 32.01% and 40.80% of patients, respectively.

**Table 2:** Treatment patterns

Name		Category	Counts	Percentage (%)	Cumulative Percentage (%)
Number of previous treatment modes		1	220	36.48	36.48
		2	291	48.26	84.74
		≥ 3	92	15.26	100.00
Previous operation		No	586	97.18	97.18
		Yes	17	2.82	100.00
Prior chemotherapy		No	196	32.50	32.50
		Yes	407	67.50	100.00
Prior radiotherapy		No	410	67.99	67.99
		Yes	193	32.01	100.00
Prior immunotherapy		No	357	59.20	59.20
		Yes	246	40.80	100.00
Previous TKI treatment		No	497	82.42	82.42
		Yes	106	17.58	100.00
Previous antivascular therapy		No	490	81.26	81.26
		Yes	113	18.74	100.00
Vaccination		No	205	34.00	34.00
		Yes	398	66.00	100.00
Total			603	100.0	100.0
Treatment of COVID-19 (*n* = 68)	Antipyretic		44	64.71	64.71
	Anti-inflammatory		14	20.59	20.59
	Traditional Chinese Medicine		6	8.82	8.82
	Steroid + Anti-inflammatory		4	5.88	5.88
Total			68	100.0	100.0

A total of 68 patients diagnosed with COVID-19 were treated with various therapies: 44 patients (64.71%) received antipyretic therapy, 14 patients (20.59%) received anti-inflammatory treatment, 6 patients (8.82%) received traditional Chinese medicine, and 4 patients (5.88%) received steroid combined with anti-inflammatory therapy. Additionally, among all patients included in the study, 398 patients (66%) were vaccinated, while 205 patients (34%) were not vaccinated.

### Analysis of high-risk factors for the occurrence of COVID-19

#### Univariate analysis of COVID-19 and comprehensive demographic

No significant associations were found between COVID-19 and sex, smoking status, or comorbidities such as hypertension, myocardial infarction, cerebral infarction, and kidney injury (*P* > 0.05, Table 3). Although age ≥ 65 years had a higher COVID-19 incidence (45.59% vs. 54.41%), the difference was not significant (*P* = 0.069). Similarly, no significant difference was found in KPS (*P* = 0.056). Cancer pathology and staging showed no significant correlation with COVID-19 infection (*P* = 0.247 and *P* = 0.256, respectively). These findings indicate that none of the analyzed factors, including demographic characteristics, comorbidities, cancer pathology, and staging, were significantly associated with COVID-19 incidence in this cohort.

**Table 3:** Univariate analysis of COVID-19 and basic information

Category	Name	COVID-19 (%)		Total	χ^2^/Z	*p*
		No	Yes			
Sex	Male	361(67.48)	46(67.65)	407(67.50)	0.001	0.977
	Female	174(32.52)	22(32.35)	196(32.50)		
Total		535	68	603		
Age	< 65 yr	351(65.61)	37(54.41)	388(64.34)	3.296	0.069
	≥ 65 yr	184(34.39)	31(45.59)	215(35.66)		
Total		535	68	603		
KPS	0 < 80	11(2.06)	4(5.88)	15(2.49)	3.641	0.056
	≥ 80	524(97.94)	64(94.12)	588(97.51)		
Total		535	68	603		
Smoke	No	311(58.13)	41(60.29)	352(58.37)	0.116	0.733
	Yes	224(41.87)	27(39.71)	251(41.63)		
Total		535	68	603		
High blood pressure	No	416(77.76)	55(80.88)	471(78.11)	0.345	0.557
	Yes	119(22.24)	13(19.12)	132(21.89)		
Total		535	68	603		
Myocardial infarction	No	521(97.38)	67(98.53)	588(97.51)	0.327	0.568
	Yes	14(2.62)	1(1.47)	15(2.49)		
Total		535	68	603		
Cerebral infarction	No	523(97.76)	67(98.53)	590(97.84)	0.171	0.680
	Yes	12(2.24)	1(1.47)	13(2.16)		
Total		535	68	603		
Kidney injury	No	534(99.81)	68(100.00)	602(99.83)	0.127	0.721
	Yes	1(0.19)	0(0.00)	1(0.17)		
Total		535	68	603		
Diabetes	No	490(91.59)	62(91.18)	552(91.54)	0.013	0.908
	Yes	45(8.41)	6(8.82)	51(8.46)		
Total		535	68	603		
Coronary heart disease	No	513(95.89)	68(100.00)	581(96.35)	2.902	0.088
	Yes	22(4.11)	0(0.00)	22(3.65)		
Total		535	68	603		
Operation History	No	523(97.76)	68(100.00)	591(98.01)	1.556	0.212
	Yes	12(2.24)	0(0.00)	12(1.99)		
Total		535	68	603		
Disease of respiratory system	No	516(96.45)	67(98.53)	583(96.68)	0.815	0.367
	Yes	19(3.55)	1(1.47)	20(3.32)		
Total		535	68	603		
Other Disease	No	517(96.64)	67(98.53)	584(96.85)	0.709	0.400
	Yes	18(3.36)	1(1.47)	19(3.15)		
Total		535	68	603		
Background disease	Without	342(63.93)	49(72.06)	391(64.84)	2.003	0.367
	1	141(26.36)	15(22.06)	156(25.87)		
	≥ 2	52(9.72)	4(5.88)	56(9.29)		
Total		535	68	603		
Pathology	Adenocarcinoma	293(54.77)	36(52.94)	329(54.56)	4.136	0.247
	Squamous carcinoma	97(18.13)	10(14.71)	107(17.74)		
	Small cell carcinoma	118(22.06)	21(30.88)	139(23.05)		
	Other carcinoma	27(5.05)	1(1.47)	28(4.64)		
Total		535(88.72)	68(11.28)	603(100.0)		
Staging	0	5(0.93)	0(0.00)	5(0.83)	5.317	0.256
	1	19(3.55)	2(2.94)	21(3.48)		
	2	18(3.36)	2(2.94)	20(3.32)		
	3	144(26.92)	27(39.71)	171(28.36)		
	4	346(65.23)	37(54.41)	383(64.01)		
Total		535	68	603		

*P < 0.05 **P < 0.01

#### Univariate analysis of COVID-19 and previous treatment patterns

In the exploration of the relationship between COVID-19 incidence and prior treatment modalities, as detailed in Table 4, the analysis revealed minimal variance in COVID-19 incidence rates between patients with a surgical history (2.94%) and those without (2.80%, χ^2^ = 0.004, *P* = 0.949). The investigation also showed that exposure to chemotherapy and radiotherapy could predispose patients to an immunocompromised state, potentially increasing their susceptibility to COVID-19. Specifically, 67.50% of the cohort (*n* = 407) received chemotherapy, 32.01% (*n* = 193) underwent radiotherapy, 40.80% (*n* = 246) received immunotherapy, and 17.58% (*n* = 106) were treated with TKIs, while 18.74% (*n* = 113) received anti-angiogenic treatment.

**Table 4:** Univariate analysis of COVID-19 and previous treatment patterns

Category	Name	COVID-19 (%)		Total	χ^2^	*P*
		No	Yes			
Previous operation	No	520(97.20)	66(97.06)	586(97.18)	0.004	0.949
	Yes	15(2.80)	2(2.94)	17(2.82)		
Total		535	68	603		
Prior chemotherapy	No	182(34.02)	14(20.59)	196(32.50)	4.960	0.026*
	Yes	353(65.98)	54(79.41)	407(67.50)		
Total		535	68	603		
Prior radiotherapy	No	374(69.91)	36(52.94)	410(67.99)	7.979	0.005**
	Yes	161(30.09)	32(47.06)	193(32.01)		
Total		535	68	603		
Prior immunotherapy	No	319(59.63)	38(55.88)	357(59.20)	0.350	0.554
	Yes	216(40.37)	30(44.12)	246(40.80)		
Total		535	68	603		
Previous TKI treatment	No	437(81.68)	60(88.24)	497(82.42)	1.788	0.181
	Yes	98(18.32)	8(11.76)	106(17.58)		
Total		535	68	603		
Previous antivascular therapy	No	434(81.12)	56(82.35)	490(81.26)	0.060	0.806
	Yes	101(18.88)	12(17.65)	113(18.74)		
Total		535	68	603		

*P < 0.05 **P < 0.01

A significant disparity in COVID-19 incidence was observed between patients who had not received chemotherapy (20.59%) and those who had (34.02%, χ^2^ = 4.960, *P* = 0.026). Additionally, COVID-19 incidence was significantly higher in patients who had undergone radiotherapy (30.09%) compared to those who had not (69.91%, χ^2^ = 7.979, *P* = 0.005). These findings indicated a notable association between chemotherapy and radiotherapy exposure and an increased susceptibility to COVID-19. In contrast, immunotherapy, prior TKI therapy, and anti-angiogenic treatment did not increase the risk of COVID-19 infection.

#### Logistic regression analysis of risk factors

In model 1, COVID-19 infection status was the dependent variable, while sex, KPS score, smoking status, and basic disease were included as independent variables (Table 5). The regression equation for the model is expressed as follows: ln(P/(1 − P)) = − 3.678 + 0.342 × Sex − 0.278 × KPS score + 0.153 × Smoking status + 1.945 × Basic disease, where P denotes the probability of COVID-19 infection (*P* = 1), and (1 - P) represents the probability of not being infected (*P* = 0). The analysis identified basic disease as a significant determinant of COVID-19 infection, with a coefficient of 1.945, yielding an OR of 6.995 (95% CI: 4.644–10.537, *P* < 0.001). This suggests that individuals with underlying medical conditions were approximately seven times more likely to contract COVID-19 than those without comorbidities. In contrast, no significant associations were found for sex, KPS score, smoking status, or previous chemotherapy and radiotherapy (all *P* > 0.05).

**Table 5:** Binary logistic regression

Category	regression coefficient	SE	Wald χ^2^	*P*	OR	OR (95% CI)
Sex	0.342	0.411	0.690	0.406	1.407	0.629 ~ 3.151
KPS score	-0.278	0.871	0.102	0.750	0.757	0.137 ~ 4.177
Smoke	0.153	0.365	0.177	0.674	1.166	0.570 ~ 2.383
Basic Disease	1.945	0.209	86.605	< 0.001	6.995	4.644 ~ 10.537
Intercept	-3.678	0.998	13.854	< 0.001	0.025	
Prior chemotherapy	0.729	0.316	5.334	0.021	2.074	1.117 ~ 3.851
Prior radiotherapy	0.759	0.262	8.357	0.004	2.136	1.277 ~ 3.572
Intercept	-2.886	0.308	87.627	< 0.001		

Dependent variable: COVID-19

In model 2, the dependent variable remained COVID-19 infection, with prior chemotherapy and radiotherapy as independent variables (Table 5). The model 2 formula is as follows: ln(P/1-P) = -2.886 + 0.729 * previous chemotherapy + 0.759 * previous radiotherapy. The regression analysis revealed that prior chemotherapy was associated with an OR of 2.074 (95% CI: 1.117–3.851, *P* = 0.021), indicating that patients with a history of chemotherapy had more than twice the likelihood of contracting COVID-19 compared to those without such a history. Similarly, prior radiotherapy was significantly associated with COVID-19 infection, with an OR of 2.136 (95% CI: 1.277–3.572, *P* = 0.004), indicating that radiotherapy exposure notably increased the odds of infection.

In the multiple logistic regression model, COVID-19 infection status was treated as the dependent variable, while pathology and staging were included as independent variables (Table 6). The analysis showed that neither pathology (*P* = 0.144, OR = 1.176, 95% CI: 0.946–1.463) nor staging (*P* = 0.050, OR = 1.491, 95% CI: 1.000–2.222) was significantly associated with COVID-19 infection (All *P* > 0.05).

**Table 6:** Multiple logistic regression

Category	regression coefficient	SE	Wald χ^2^	*P*	OR	OR (95% CI)
Pathology	0.162	0.111	2.131	0.144	1.176	0.946 ~ 1.463
Staging	0.399	0.204	3.846	0.050	1.491	1.000 ~ 2.222
Intercept	-3.812	0.811	21.987	< 0.001		

Dependent variable: COVID-19

### Analysis of the influence of infection with novel coronavirus pneumonia on treatment

Table 7 summarizes the suspension of cancer treatments during the COVID-19 pandemic. Surgical interventions were largely unaffected, with 87.50% (*n* = 14 / 16) proceeding as planned. In contrast, chemotherapy faced significant disruptions, as 42.86% (*n* = 168 / 392) of treatments were suspended. Radiotherapy displayed a mixed response, with 52.38% (*n* = 99 / 189) continuing, while 47.62% (*n* = 90) were interrupted. Immunotherapy treatments showed greater continuity, with 60.59% (*n* = 143 / 236) ongoing, compared to 39.41% (*n* = 93) that were paused. TKI therapy exhibited notable resilience, with 80.20% (*n* = 81 / 101) of patients maintaining their schedules. Anti-vascular therapy experienced a near-equal distribution, as 50.93% (*n* = 55 / 108) continued, while 49.07% (*n* = 53) were halted.

**Table 7:** Status of suspension due to COVID-19 under different treatment modes

Category	Treatment suspension	Counts	Percentage (%)	Cumulative percentage (%)
Previous surgery (*n* = 16)	No	14	87.50	87.50
	Yes	2	12.50	100.00
Previous chemotherapy (*n* = 392)	No	224	57.14	57.14
	Yes	168	42.86	100.00
Previous radiotherapy (*n* = 189)	No	99	52.38	52.38
	Yes	90	47.62	100.00
Previous immunotherapy (*n* = 236)	No	143	60.59	60.59
	Yes	93	39.41	100.00
Previous TKI treatment (*n* = 101)	No	81	80.20	80.20
	Yes	20	19.80	100.00
Previous antivascular therapy (*n* = 108)	No	55	50.93	50.93
	Yes	53	49.07	100.00

### The difference of restart time interval between different treatment modes

Table 8 presents the Kruskal-Wallis test results comparing restart intervals across six treatment types, revealing significant differences (H = 13.314, *P* = 0.021). TKI treatment had the shortest restart interval, with a median of 1 day, though the sample size limited its applicability. Immunotherapy resumed after a median delay of 12 days, with a range of 5 to 22 days. Chemotherapy restarted faster, with a median delay of 9 days and a range of 4 to 16.3 days. Surgical treatments showed variability, with resumption times ranging from 1 to 20.5 days, and a median of 1 day. Anti-angiogenic therapies had the longest median delay of 35 days, with a range of 22 to 48 days.

**Table 8:** Differences in restart intervals between the 6 previous treatment modes

Prior treatment	Restart interval
Previous TKI treatment (*n* = 1)	1.0(1.0,1.0)
Previous immunotherapy (*n* = 39)	12.0(5.0,22.0)
Previous chemotherapy (*n* = 86)	9.0(4.0,16.3)
Previous surgery (*n* = 4)	1.0(1.0,20.5)
Previous antivascular therapy (*n* = 2)	35.0(22.0,48.0)
Previous radiotherapy (*n* = 16)	3.5(1.0,23.5)
Kruskal-Wallis test statistic H value	13.31
*P*	0.021*

*P < 0.05 **P < 0.01

In conclusion, the analysis highlighted significant variability in resumption times, with anti-angiogenic therapy experiencing the longest delays, while TKI therapy and surgery resumed more promptly.

### Variation in efficacy evaluation and toxicity evaluation after 2 cycles with or without treatment suspension

#### Difference in efficacy evaluation after 2 cycles with or without suspension of treatment

Table 9 examines the impact of COVID-19-related treatment suspensions on efficacy after two cycles. In the uninterrupted treatment group, 0.45% of cases achieved CR, while no CR was observed in the suspended treatment group, though this difference was not statistically significant. PD occurred in 8.11% of the uninterrupted group and 6.70% of the suspended group. PR rates were similar, at 4.95% for the uninterrupted group and 4.47% for the suspended group. SD was the most common outcome in both groups, with 86.49% in the non-suspended group and 88.83% in the suspended group. These results indicate that COVID-19-related treatment suspensions did not significantly affect treatment efficacy upon resumption (χ^2^ = 1.179, *P* = 0.758), with the majority of patients showing stable disease in both groups.

**Table 9:** Differences in efficacy evaluation after 2 cycles with or without suspension of treatment

Category	Name	Whether treatment was suspended due to COVID-19 (%)		Total	χ^2^	*P*
		No	Yes			
Evaluation of curative effect after 2 cycles	CR	1(0.45)	0(0.00)	1(0.25)	1.179	0.758
	PD	18(8.11)	12(6.70)	30(7.48)		
	PR	11(4.95)	8(4.47)	19(4.74)		
	SD	192(86.49)	159(88.83)	351(87.53)		
Total		222	179	401		

*P < 0.05 **P < 0.01

#### Differences in evaluation of toxicity with or without suspension of treatment

Fatigue occurred at similar rates across both cohorts, with 6.98% in the ongoing treatment group and 6.06% in the suspended treatment group, indicating that treatment interruption did not significantly affect fatigue prevalence (Table 10). Electrolyte disturbances were observed exclusively in the suspended treatment group, at a rate of 9.09%. Dermatitis was rare, affecting 3.03% of the suspended group and was absent in the uninterrupted group. Secondary pneumonia rates were comparable between the groups, with 13.95% in the continuous treatment group and 12.12% in the suspended group. Hepatic or renal impairment was reported by 4.65% of the non-suspended group and 3.03% of the suspended group. Gastrointestinal side effects were more prevalent in the non-suspended group (16.28%) compared to the suspended group (3.03%), suggesting a stronger association with continuous treatment. Bone marrow suppression emerged as the most common toxicity, with a slightly higher incidence in the suspended group (63.64%) compared to the non-suspended group (58.14%). In conclusion, the interruption of cancer treatments due to the COVID-19 pandemic did not significantly alter the overall toxicity profile of these treatments (χ^2^ = 1.179, *P* = 0.758).

**Table 10:** Differences in toxicity evaluation of treatment suspension

Category	Name	Suspension of treatment due to COVID-19 (%)		Total	χ^2^	*p*
		No	Yes			
Toxicity assessment	Feeble	3(6.98)	2(6.06)	5(6.58)	8.615	0.196
	Electrolyte disturbance	0(0.00)	3(9.09)	3(3.95)		
	Dermatitis	0(0.00)	1(3.03)	1(1.32)		
	Secondary pneumonia	6(13.95)	4(12.12)	10(13.16)		
	Hepatic and renal impairment	2(4.65)	1(3.03)	3(3.95)		
	Gastrointestinal reaction	7(16.28)	1(3.03)	8(10.53)		
	Myelosuppression	25(58.14)	21(63.64)	46(60.53)		
Total		43	33	76		

*P < 0.05 **P < 0.01

### The difference of efficacy evaluation and toxicity evaluation after 2 cycles of different restart treatment modes

#### The difference of efficacy evaluation after 2 cycles of different restart treatment modes

Upon resumption of treatments, an analysis revealed that CR was exceedingly rare across all treatment types, occurring only in immunotherapy and chemotherapy, with rates of 0.66% and 0.39%, respectively (Table 11). In contrast, PD rates varied widely, with the highest observed in TKI therapy (19.05%) and anti-angiogenic therapy (14.06%), and the lowest in radiotherapy (7.37%). No partial response (PR) was noted in TKI therapy and surgery, while immunotherapy and chemotherapy showed PR rates of 5.92% and 5.10%. The most frequent outcome was stable disease (SD), which was observed across all treatment types, with 100% SD in surgery and over 86% in both immunotherapy and chemotherapy. These findings suggest that, although complete or partial responses were infrequent, the majority of patients achieved disease stability after treatment resumption.

**Table 11:** Differences in efficacy evaluation after 2 cycles of different restart treatment modes

Category	Name	Restart Healing mode (%)						Total	χ^2^	*P*
		TKI treatment	Immunotherapy	Chemotherapy	Operation	Anti-angiogenic therapy	Radiotherapy			
Evaluation of post-cycle efficacy	CR	0(0.00)	1(0.66)	1(0.39)	0(0.00)	0(0.00)	0(0.00)	2(0.33)	13.420	0.570
	PD	8(19.05)	11(7.24)	21(8.24)	0(0.00)	9(14.06)	7(7.37)	56(9.15)		
	PR	0(0.00)	9(5.92)	13(5.10)	0(0.00)	1(1.56)	5(5.26)	28(4.58)		
	SD	34(80.95)	131(86.18)	220(86.27)	4(100.00)	54(84.38)	83(87.37)	526(85.95)		
Total		42	152	255	4	64	95	612		

*P < 0.05 **P < 0.01

#### The difference of toxicity evaluation in different reactivation therapy modes

A comparative analysis of the toxicity profiles following treatment resumption in cancer patients after COVID-19 infection revealed minor variations across different cancer treatments (Table 12). Fatigue was absent in TKI therapy and surgery, while anti-angiogenic therapy exhibited the highest incidence at 12.50%. Dermatitis was observed in immunotherapy and radiotherapy, with incidences of 5.00% and 4.76%, respectively. Hepatic or renal impairment was more frequently reported in immunotherapy and anti-angiogenic therapy, with rates of 10.00% and 12.50%, while no such impairments were noted in TKI therapy, surgery, or radiotherapy. Gastrointestinal reactions were more pronounced in chemotherapy and immunotherapy, with incidences of 11.54% and 10.00%, respectively. Bone marrow suppression was the most prevalent toxicity across all treatment modalities, particularly in chemotherapy, immunotherapy, and radiotherapy, with reported rates of 65.38%, 60.00%, and 52.38%, respectively. In conclusion, bone marrow suppression emerged as the most common adverse effect across nearly all treatment types, most notably in chemotherapy, immunotherapy, and radiotherapy.

**Table 12:** Differences in toxicity evaluation of different reactivation therapy modes

Category	Name	Restart Healing mode (%)						Total	χ^2^	*P*
		TKI treatment	Immunotherapy	Chemotherapy	operation	Antivascular therapy	Radiotherapy			
Toxicity assessment	Lacking in strength	0(0.00)	1(5.00)	3(5.77)	0(0.00)	1(12.50)	1(4.76)	6(5.77)	25.778	0.686
	Electrolyte disturbance	0(0.00)	0(0.00)	1(1.92)	0(0.00)	0(0.00)	3(14.29)	4(3.85)		
	Dermatitis	0(0.00)	1(5.00)	0(0.00)	0(0.00)	0(0.00)	1(4.76)	2(1.92)		
	Secondary pneumonia	1(50.00)	2(10.00)	6(11.54)	1(100.00)	2(25.00)	4(19.05)	16(15.38)		
	Liver and kidney function is impaired	0(0.00)	2(10.00)	2(3.85)	0(0.00)	1(12.50)	0(0.00)	5(4.81)		
	Gastrointestinal reaction	0(0.00)	2(10.00)	6(11.54)	0(0.00)	0(0.00)	1(4.76)	9(8.65)		
	Myelosuppression	1(50.00)	12(60.00)	34(65.38)	0(0.00)	4(50.00)	11(52.38)	62(59.62)		
Total		2	20	52	1	8	21	104		

*P < 0.05 **P < 0.01

## Discussion

### Cancer, complications and COVID-19

Cancer patients are at increased risk for severe SARS-CoV-2 infection due to their immunocompromised status, which is a consequence of both the malignancy itself and the treatments they receive [16]. This heightened vulnerability is further exacerbated by frequent healthcare visits during active cancer treatment, as well as factors such as compromised immune function and complications arising from anti-cancer therapies [17]. A study assessing the seroprevalence of SARS-CoV-2 IgG antibodies in cancer patients prior to vaccination revealed that 29% of patients had detectable antibodies, including 20% without a documented history of COVID-19 infection [18]. The seroconversion rate for patients with a confirmed history of COVID-19 was 79.5%, which was notably lower than that observed in the general population. These findings suggest that cancer patients may exhibit a diminished immune response to SARS-CoV-2, thereby increasing their susceptibility to severe COVID-19 outcomes. According to the investigation, the COVID-19 positivity rate was likely influenced by cancer stage, type, and overall health status, including comorbidities such as age, race, ethnicity, smoking habits, and body mass index [19–21].

This study included 603 cancer patients, of whom 68 (11.28%) were infected with COVID-19, 398 (66%) were vaccinated, and 205 (34%) were unvaccinated. Regarding vaccine safety, this study found no severe adverse reactions to the vaccine. Studies have shown that the inactivated SARS-CoV-2 vaccine is safe and effective in cancer patients, with injection site pain and fever being the most common side effects [22, 23]. The overall seroconversion rate was 86.9%, with lower rates observed in older patients, those with hematological malignancies, and those undergoing chemotherapy [22]. In addition to age and comorbidities, smoking status was also identified as a potential risk factor for severe COVID-19 in cancer patients [19–21]. In this study, 58.37% of patients were non-smokers, and 41.63% were smokers. Unfortunately, the regression analysis did not demonstrate a significant association between age, sex, and smoking history with COVID-19 infection in this cancer cohort.

Although this study did not find a significant association between cancer type, staging, or KPS with COVID-19 incidence in the cohort, other studies have reported such correlations. For instance, a case report highlighted that patients with advanced cancer, particularly those with Stage IV thoracic esophageal cancer, were more susceptible to severe COVID-19 infections, which could lead to treatment interruptions [24]. Notably, the most common cancer type in this cohort was adenocarcinoma (54.56%), and patients with more advanced cancer, especially Stage IV (64.01%), were found to be more vulnerable to COVID-19, as reported in previous studies [12, 25].

The binary logistic regression analysis revealed that cancer patients with underlying conditions were more likely to contract COVID-19. The investigation indicated that cancer and cardiovascular diseases share common risk factors, such as hypertension, diabetes, and obesity [26]. Studies have shown that patients with these conditions are more likely to develop COVID-19 and experience more severe illness [16, 27, 28]. In a Chinese study of 138 hospitalized COVID-19 patients, 31.1% had hypertension and 14.1% had cardiovascular disease. Patients with these comorbidities were more likely to require ICU care [29]. A meta-analysis of 1,527 patients found that hypertension was the most common comorbidity, followed by cardiovascular disease and diabetes. Having these conditions increased the risk of ICU admission by two to three times [30]. These conditions significantly exacerbated COVID-19 severity and mortality, as seen in previous studies [31–34]. In this cohort, 21.89% of patients had hypertension, and 8.46% had diabetes. Consistent with previous studies [27], patients with cancer and cardiovascular comorbidities were more likely to develop myocarditis after contracting COVID-19 (data not shown in the table). Study highlights that diabetic individuals are more susceptible to severe COVID-19 due to altered metabolic programming in the kidney [35]. Diabetic-like kidney organoids showed higher viral loads after SARS-CoV-2 infection, and diabetic patient cells exhibited enhanced glycolysis and altered mitochondrial respiration, increasing viral susceptibility [35].

### Cancer treatment patterns and COVID-19

A study demonstrated that cancer patients receiving palliative treatments and radiotherapy were at a higher risk of contracting COVID-19, with these factors also contributing to increased mortality [6]. The results of my study align with these findings, revealing that chemotherapy induces immunosuppression, thereby elevating the risk of serious infections and complications in COVID-19. Logistic regression analysis highlighted a significant association between prior chemotherapy, radiotherapy, and the incidence of COVID-19. Chemotherapy patients are particularly vulnerable to respiratory syndromes induced by chemotherapeutic agents and are more susceptible to viral infections due to the immunosuppressive effects of treatment. A review and meta-analysis assessing the impact of COVID-19 on cancer patients reported a higher likelihood of severe outcomes and increased mortality in this population [36, 37]. Interestingly, during the COVID-19 epidemic control period, the infection rate in patients with Hodgkin lymphoma receiving chemotherapy decreased to 2.1% of their treatment cycles, accounting for 24.1% of total cases, suggesting a reduction in the overall infection rate [38]. While recent chemotherapy treatments were not directly associated with worse COVID-19 outcomes, specific factors, such as active hematologic or lung malignancies and baseline neutropenia, were found to exacerbate outcomes [39].

Similarly, radiation therapy targeting the chest region may impair lung function, thereby worsening the impact of respiratory infections like COVID-19. No statistically significant associations were observed between surgery, immunotherapy, TKI therapy, and anti-angiogenic therapy with COVID-19 incidence. The low incidence of surgery among the cohort may reflect the limited extent of invasive procedures, which could be beneficial in the context of COVID-19, as surgical histories may complicate respiratory illnesses [40]. Furthermore, studies have indicated that patients with adenocarcinoma who received immunotherapy within four weeks prior to COVID-19 diagnosis had a reduced risk of death [41]. These findings suggest that the interactions between antineoplastic therapy, cancer type, and COVID-19 outcomes are complex and warrant further investigation under specific clinical conditions [41].

### Adjustment and efficacy of cancer treatment modalities

Studies on the impact of chemotherapy and immunotherapy have confirmed that recent chemotherapy treatments are a strong risk factor for inadequate antibody responses, while immunotherapy may have a more limited effect on vaccine efficacy [42–44]. Regarding treatment modalities, radiotherapy has been adapted during the pandemic to replace or delay higher-infection-risk treatments [36]. For instance, short courses of radiotherapy have been used to delay surgery in certain cancer cases. However, with increasing demand, there may be a need to ration radiotherapy [36]. The risk associated with biological and monoclonal-antibody therapies is less clear, and some might be beneficial against COVID-19’s inflammatory storm. Clinical guidelines have been published to inform the systemic treatment of cancer during the pandemic, taking into account the balance of risks and benefist [36]. However, it’s important to note that recent treatment, including immunotherapy, does not significantly impact the outcome of COVID-19 in cancer patients [45]. The variation in treatment suspension rates across different modalities suggests that the decision to continue or halt treatment was not uniform but rather dependent on the type of treatment and possibly the patient’s overall health status and COVID-19 severity. Particularly, patients undergoing treatments that could potentially compromise their immune system, such as chemotherapy, might have been more prone to treatment suspensions due to heightened risks associated with COVID-19 infection.

These disruptions in care also led to a greater need for post-surgical radiation therapy, indicating worsened clinical outcomes [46]. Previous studies have suggested that COVID-19 may impact cancer patients differently based on disease stage and performance status, with certain cancer types showing varying susceptibility to infection and outcomes [47]. Specifically, the multivariate Cox regression analysis revealed that lung cancer (HR 1.76, CI95 1.23–2.53, *p* < 0.05), stage III (HR 3.63, CI95 2.21–5.98, *p* < 0.05), stage IV (HR 11.06, CI95 7.04–17.36, *p* < 0.05), and age at diagnosis (HR 1.04, CI95 1.02–1.05, *p* < 0.05) were independent predictors of poorer OS [47]. Especially due to treatment delays caused by the COVID-19 lockdown, there was a significant increase in tumor size, more advanced N-staging, and a higher incidence of extreme advanced disease in breast cancer patients [46, 48].

### Unanswered Questions in COVID-19 and cancer research

Studies have shown that COVID-19 mRNA vaccines are safe for cancer patients and can induce seroconversion, although response rates are lower in those with hematologic malignancies or undergoing chemotherapy [49, 50]. The majority of solid tumor patients are able to seroconvert after two vaccine doses, and a third dose significantly improves immunization rates [50–52]. Given these findings, it is recommended that all cancer patients receive the full vaccination regimen, with a potential third booster dose for those actively undergoing chemotherapy or suffering from hematologic cancers [49]. Adverse events from vaccination are generally mild and include symptoms like pain at the injection site, fatigue, and headaches, though there are concerns regarding immune-related adverse events (irAEs) in patients undergoing immune checkpoint inhibitors and potential radiation reactions post-vaccination [53, 54].

Several key questions remain unanswered in clinical COVID-19 and cancer research. The effects of SARS-CoV-2 on lung cancer pathogenesis, particularly whether it directly infects cancer cells and influences tumor progression, remain unclear. Additionally, the long-term consequences of COVID-19 vaccines on immune function in cancer patients, especially those receiving immunosuppressive therapies, need further exploration. The impact of emerging COVID-19 variants, such as Delta and Omicron, on cancer patients’ immune responses and outcomes post-vaccination is also under-researched [55, 56]. Furthermore, the interaction between cancer therapies, including immune checkpoint inhibitors, and vaccine efficacy requires a deeper understanding.

Future research should focus on longitudinal, multi-center studies to evaluate the durability of vaccine protection and its interaction with different treatment regimens. The effectiveness of vaccines against emerging variants should be closely monitored, and the use of heterologous booster vaccines warrants further investigation. Additionally, more research is needed to understand the direct effects of SARS-CoV-2 infection on tumor progression and the combined impact of infection and vaccination on cancer treatment outcomes. Addressing these gaps is crucial to optimizing the management of cancer patients during the ongoing pandemic and future health crises.

## Conclusion

COVID-19 incidence was low in the patient cohort, with no significant associations found between gender, age, or common comorbidities such as hypertension, diabetes, or cardiovascular diseases. However, patients undergoing chemotherapy and radiotherapy exhibited a significantly higher incidence of COVID-19. Binary logistic regression analysis further revealed that chemotherapy and radiotherapy were risk factors for COVID-19 infection, and patients with underlying diseases were more prone to infection. A considerable proportion of the cohort experienced treatment interruptions due to the pandemic, particularly those receiving chemotherapy, radiotherapy, and anti-angiogenic therapy. Treatment resumption did not show significant differences in efficacy or toxicity profiles between patients with and without treatment suspension, with Stable Disease being the most common outcome across all modalities. These findings emphasize the increased susceptibility of patients receiving chemotherapy and radiotherapy to COVID-19 and underscore the importance of tailored management strategies for these high-risk groups.

## Data Availability

Data is provided within the manuscript.

## Abbreviations

COVID-19: Corona virus disease 2019
SARS-CoV-2: Severe acute respiratory syndrome corona virus 2
RT-PCR: Quantitative reverse transcription polymerase chain reaction
KPS: Karnofsky performance status
CR: Complete response
PR: Partial response
SD: Stable disease
PD: Progressive disease

## References

[1] Lu R, Zhao X, Li J, Niu P, Yang B, Wu H, et al. Genomic characterisation and epidemiology of 2019 novel coronavirus: implications for virus origins and receptor binding. Lancet. 2020;395(10224):565–74. 32007145 10.1016/S0140-6736(20)30251-8 PMC7159086

[2] Guan WJ, Ni ZY, Hu Y, Liang WH, Ou CQ, He JX, et al. Clinical characteristics of coronavirus disease 2019 in China. N Engl J Med. 2020;382(18):1708–20. 32109013 10.1056/NEJMoa2002032 PMC7092819

[3] Conhecimento RDIE, Coronavirus. COVID-19 Global Cases by the Center for Systems Science and Engineering (CSSE). 2020.

[4] Coelho DB, Santos V, Araújo D, Bastos HN, Magalhães A, Hespanhol V, et al. Management of coronavirus disease 2019 patients with lung cancer: experience from a thoracic oncology center. Front Mol Biosci. 2021;8:639676. 34368223 10.3389/fmolb.2021.639676 PMC8339998

[5] Soroosh D, Javadinia SA. The COVID-19 Outbreak and Oncology Centers in Iran. Int J Cancer Manag. 13(6).

[6] Fazilat-Panah D, Fallah Tafti H, Rajabzadeh Y, Fatemi MA, Ahmadi N, Jahansouz D, et al. Clinical characteristics and outcomes of COVID-19 in 1294 new Cancer patients: Single-Center, prospective cohort study from Iran. Cancer Invest. 2022;40(6):505–15. 35521692 10.1080/07357907.2022.2075376

[7] Tian Kun L, Yating Z, Bei W, Tao C, Tianxing P, Xiaxia et al. Immunological status and treatment strategies in cancer patients with COVID-19. Chin J Clin Oncol. 2022;17(049).

[8] Shahidsales S, Aledavood SA, Joudi M, Molaie F, Esmaily H, Javadinia SA. COVID-19 in cancer patients May be presented by atypical symptoms and higher mortality rate, a case-controlled study from Iran. Cancer Rep (Hoboken). 2021;4(5):e1378. 33742793 10.1002/cnr2.1378 PMC8250318

[9] Taghizadeh-Hesary F, Porouhan P, Soroosh D, PeyroShabany B, Shahidsales S, Keykhosravi B et al. COVID-19 in Cancer and Non-cancer Patients. Int J Cancer Manag. 14(4).

[10] Shen K, Yang Y, Wang T, Zhao D, Jiang Y, Jin R, et al. Diagnosis, treatment, and prevention of 2019 novel coronavirus infection in children: experts’ consensus statement. World J Pediatr. 2020;16(3):223–31. 32034659 10.1007/s12519-020-00343-7 PMC7090771

[11] Huang C, Wang Y, Li X, Ren L, Zhao J, Hu Y, et al. Clinical features of patients infected with 2019 novel coronavirus in Wuhan, China. Lancet. 2020;395(10223):497–506. 31986264 10.1016/S0140-6736(20)30183-5 PMC7159299

[12] Pan S, Jiang J, Chen Z, Yang L. Management and thinking on the treatment of Cancer patients during the COVID-19. Front Mol Biosci. 2021;8:673360. 34277701 10.3389/fmolb.2021.673360 PMC8282485

[13] Tian J, Yuan X, Xiao J, Zhong Q, Yang C, Liu B, et al. Clinical characteristics and risk factors associated with COVID-19 disease severity in patients with cancer in Wuhan, China: a multicentre, retrospective, cohort study. Lancet Oncol. 2020;21(7):893–903. 32479790 10.1016/S1470-2045(20)30309-0 PMC7259911

[14] Xu M, Wang D, Wang H, Zhang X, Liang T, Dai J, et al. COVID-19 diagnostic testing: technology perspective. Clin Transl Med. 2020;10(4):e158. 32898340 10.1002/ctm2.158 PMC7443140

[15] Kattan J, Kattan C, Assi T. Do checkpoint inhibitors compromise the cancer patients’ immunity and increase the vulnerability to COVID-19 infection? Immunotherapy. 2020;12(6):351–4. 32290754 10.2217/imt-2020-0077 PMC7161588

[16] Jyotsana N, King MR. The impact of COVID-19 on Cancer risk and treatment. Cell Mol Bioeng. 2020;13(4):285–91. 32837583 10.1007/s12195-020-00630-3 PMC7323371

[17] Alaeddini M, Etemad-Moghadam S. SARS-Cov-2 infection in cancer patients, susceptibility, outcome and care. Am J Med Sci. 2022;364(5):511–20. 35605680 10.1016/j.amjms.2022.05.017 PMC9119956

[18] Javadinia SA, Ariamanesh M, Nabavifard M, Porouhan P, PeyroShabany B, Fazilat-Panah D, et al. Multicenter study of antibody Seroprevalence against COVID-19 in patients presenting to Iranian Cancer centers after one year of the COVID-19 pandemic. Cancer Invest. 2022;40(2):115–23. 34699294 10.1080/07357907.2021.1995742

[19] Lièvre A, Turpin A, Ray-Coquard I, Le Malicot K, Thariat J, Ahle G, et al. Risk factors for coronavirus disease 2019 (COVID-19) severity and mortality among solid cancer patients and impact of the disease on anticancer treatment: A French nationwide cohort study (GCO-002 CACOVID-19). Eur J Cancer. 2020;141:62–81. 33129039 10.1016/j.ejca.2020.09.035 PMC7543792

[20] Sengar M, Chinnaswamy G, Ranganathan P, Ashok A, Bhosale S, Biswas S, et al. Outcomes of COVID-19 and risk factors in patients with cancer. Nat Cancer. 2022;3(5):547–51. 35379984 10.1038/s43018-022-00363-4

[21] Alizadehsani R, Alizadeh Sani Z, Behjati M, Roshanzamir Z, Hussain S, Abedini N, et al. Risk factors prediction, clinical outcomes, and mortality in COVID-19 patients. J Med Virol. 2021;93(4):2307–20. 33247599 10.1002/jmv.26699 PMC7753243

[22] Ariamanesh M, Porouhan P, PeyroShabany B, Fazilat-Panah D, Dehghani M, Nabavifard M, et al. Immunogenicity and safety of the inactivated SARS-CoV-2 vaccine (BBIBP-CorV) in patients with malignancy. Cancer Invest. 2022;40(1):26–34. 34634986 10.1080/07357907.2021.1992420 PMC8567287

[23] Javadinia SA, Alizadeh K, Mojadadi MS, Nikbakht F, Dashti F, Joudi M, et al. COVID-19 vaccination in patients with malignancy; A systematic review and Meta-Analysis of the efficacy and safety. Front Endocrinol (Lausanne). 2022;13:860238. 35586627 10.3389/fendo.2022.860238 PMC9108702

[24] Umezu M, Takeno A, Hamakawa T, Toshiyama R, Kawai K, Takahashi Y, et al. [A case of thoracic esophageal Cancer treated with COVID-19 pneumonia during preoperative Chemoradiotherapy(CRT)]. Gan Kagaku Ryoho. 2023;50(2):267–9. 36807193

[25] Verma R, Foster RE, Horgan K, Mounsey K, Nixon H, Smalle N, et al. Lymphocyte depletion and repopulation after chemotherapy for primary breast cancer. Breast Cancer Res. 2016;18(1):10. 26810608 10.1186/s13058-015-0669-x PMC4727393

[26] Koene RJ, Prizment AE, Blaes A, Konety SH. Shared risk factors in cardiovascular disease and Cancer. Circulation. 2016;133(11):1104–14. 26976915 10.1161/CIRCULATIONAHA.115.020406 PMC4800750

[27] Palaskas NL, Koutroumpakis E, Deswal A. COVID-19 and cardiovascular health among patients with Cancer. Curr Cardiol Rep. 2020;22(12):171. 33040241 10.1007/s11886-020-01421-y PMC7547820

[28] de Las Heras B, Saini KS, Boyle F, Ades F, de Azambuja E, Bozovic-Spasojevic I, et al. Cancer treatment and research during the COVID-19 pandemic: experience of the first 6 months. Oncol Ther. 2020;8(2):171–82. 32749634 10.1007/s40487-020-00124-2 PMC7402077

[29] Wang D, Hu B, Hu C, Zhu F, Liu X, Zhang J, et al. Clinical characteristics of 138 hospitalized patients with 2019 novel Coronavirus-Infected pneumonia in Wuhan, China. JAMA. 2020;323(11):1061–9. 32031570 10.1001/jama.2020.1585 PMC7042881

[30] Li B, Yang J, Zhao F, Zhi L, Wang X, Liu L, et al. Prevalence and impact of cardiovascular metabolic diseases on COVID-19 in China. Clin Res Cardiol. 2020;109(5):531–8. 32161990 10.1007/s00392-020-01626-9 PMC7087935

[31] Bartoloni E, Perricone C, Cafaro G, Gerli R. Hypertension and SARS-CoV-2 infection: is inflammation the missing link? Cardiovasc Res. 2020;116(13):e193–4. 32966547 10.1093/cvr/cvaa273 PMC7543278

[32] Zheng YY, Ma YT, Zhang JY, Xie X. COVID-19 and the cardiovascular system. Nat Reviews Cardiol. 2020;17(5):1–2. 10.1038/s41569-020-0360-5 PMC7095524 32139904

[33] Guzik TJ, Mohiddin SA, Dimarco A, Patel V, Savvatis K, Marelli-Berg FM, et al. COVID-19 and the cardiovascular system: implications for risk assessment, diagnosis, and treatment options. Cardiovasc Res. 2020;116(10):1666–87. 32352535 10.1093/cvr/cvaa106 PMC7197627

[34] Zhu L, She ZG, Cheng X, Qin JJ, Zhang XJ, Cai J, et al. Association of blood glucose control and outcomes in patients with COVID-19 and Pre-existing type 2 diabetes. Cell Metab. 2020;31(6):1068–e773. 32369736 10.1016/j.cmet.2020.04.021 PMC7252168

[35] Garreta E, Prado P, Stanifer ML, Monteil V, Marco A, Ullate-Agote A, et al. A diabetic milieu increases ACE2 expression and cellular susceptibility to SARS-CoV-2 infections in human kidney organoids and patient cells. Cell Metab. 2022;34(6):857–e739. 35561674 10.1016/j.cmet.2022.04.009 PMC9097013

[36] Richards M, Anderson M, Carter P, Ebert BL, Mossialos E. The impact of the COVID-19 pandemic on cancer care. Nat Cancer. 2020;1(6):565–7. 35121972 10.1038/s43018-020-0074-y PMC7238956

[37] Venkatesulu BP, Chandrasekar VT, Girdhar P, Advani P, Sharma A, Elumalai T, et al. A systematic review and meta-analysis of cancer patients affected by a novel coronavirus. MedRxiv. 2020 May 29:2020.05.27.20115303. 10.1093/jncics/pkaa102 PMC7928783 33875976

[38] Jacob AS, Kaul H, Fuchs M, Gillessen S, Kreissl S, Pluetschow A, et al. Impact of the first COVID-19 lockdown in Germany on the rate of acute infections during intensive chemotherapy for hodgkin lymphoma. Infection. 2022;50(4):925–32. 35182355 10.1007/s15010-022-01765-3 PMC8857743

[39] Jee J, Foote MB, Lumish M, Stonestrom AJ, Wills B, Narendra V, et al. Chemotherapy and COVID-19 outcomes in patients with Cancer. J Clin Oncol. 2020;38(30):3538–46. 32795225 10.1200/JCO.20.01307 PMC7571792

[40] Williams M, Mi E, Le Calvez K, Chen J, Pakzad-Shahabi L, Dadhania S, et al. Estimating the risk of death from COVID-19 in adult Cancer patients. Clin Oncol (R Coll Radiol). 2021;33(3):e172–9. 33218850 10.1016/j.clon.2020.10.021 PMC7671644

[41] Várnai C, Palles C, Arnold R, Curley HM, Purshouse K, Cheng VWT, et al. Mortality among adults with Cancer undergoing chemotherapy or immunotherapy and infected with COVID-19. JAMA Netw Open. 2022;5(2):e220130. 35188551 10.1001/jamanetworkopen.2022.0130 PMC8861846

[42] Fendler A, Shepherd STC, Au L, Wilkinson KA, Wu M, Byrne F, et al. Adaptive immunity and neutralizing antibodies against SARS-CoV-2 variants of concern following vaccination in patients with cancer: the CAPTURE study. Nat Cancer. 2021;2(12):1305–20. 35121899 10.1038/s43018-021-00274-w

[43] Peeters M, Verbruggen L, Teuwen L, Vanhoutte G, Vande Kerckhove S, Peeters B, et al. Reduced humoral immune response after BNT162b2 coronavirus disease 2019 messenger RNA vaccination in cancer patients under antineoplastic treatment. ESMO Open. 2021;6(5):100274. 34597941 10.1016/j.esmoop.2021.100274 PMC8423808

[44] Rousseau B, Loulergue P, Mir O, Krivine A, Kotti S, Viel E, et al. Immunogenicity and safety of the influenza A H1N1v 2009 vaccine in cancer patients treated with cytotoxic chemotherapy and/or targeted therapy: the VACANCE study. Ann Oncol. 2012;23(2):450–7. 21576285 10.1093/annonc/mdr141

[45] Fillmore NR, La J, Szalat RE, Tuck DP, Nguyen V, Yildirim C, et al. Prevalence and outcome of COVID-19 infection in Cancer patients: A National veterans affairs study. J Natl Cancer Inst. 2021;113(6):691–8. 33031532 10.1093/jnci/djaa159 PMC7665587

[46] Vanni G, Pellicciaro M, Materazzo M, Pedini D, Portarena I, Buonomo C, et al. Advanced stages and increased need for adjuvant treatments in breast Cancer patients: the effect of the One-year COVID-19 pandemic. Anticancer Res. 2021;41(5):2689–96. 33952500 10.21873/anticanres.15050

[47] Kohan A, Menon S, Murad V, Mirshahvalad SA, Kulanthaivelu R, Farag A et al. Impact of the COVID-19 pandemic on staging oncologic PET/CT imaging and patient outcome in a public healthcare context: overview and follow up of the first two years of the pandemic. Cancers (Basel). 2023;15(22). 10.3390/cancers15225358 PMC10670509 38001619

[48] Garip H, Baskonus I, Aytekin A, Yilmaz L, Bulut A, Gumus M. The effect of the COVID-19 pandemic on clinical and pathologic stages of patients diagnosed with breast cancer. Ir J Med Sci. 2025;194(1):37–44. 39754685 10.1007/s11845-024-03860-w

[49] Javadinia SA, Welsh JS, Mosavi Jarrahi A. COVID-19 vaccination and cancer, the need for more data. Asian Pac J Cancer Prev. 2021;22(10):3053–4. 34710978 10.31557/APJCP.2021.22.10.3053 PMC8858232

[50] Trivanović D, Peršurić Ž, Agaj A, Jakopović M, Samaržija M, Bitar L et al. The interplay of lung cancer, COVID-19, and vaccines. Int J Mol Sci. 2022;23(23). 10.3390/ijms232315067 PMC9738445 36499394

[51] Thakkar A, Gonzalez-Lugo JD, Goradia N, Gali R, Shapiro LC, Pradhan K, et al. Seroconversion rates following COVID-19 vaccination among patients with cancer. Cancer Cell. 2021;39(8):1081–e902. 34133951 10.1016/j.ccell.2021.06.002 PMC8179248

[52] Monin L, Laing AG, Muñoz-Ruiz M, McKenzie DR, Del Molino Del Barrio I, Alaguthurai T, et al. Safety and immunogenicity of one versus two doses of the COVID-19 vaccine BNT162b2 for patients with cancer: interim analysis of a prospective observational study. Lancet Oncol. 2021;22(6):765–78. 33930323 10.1016/S1470-2045(21)00213-8 PMC8078907

[53] Waissengrin B, Agbarya A, Safadi E, Padova H, Wolf I. Short-term safety of the BNT162b2 mRNA COVID-19 vaccine in patients with cancer treated with immune checkpoint inhibitors. Lancet Oncol. 2021;22(5):581–3. 33812495 10.1016/S1470-2045(21)00155-8 PMC8016402

[54] Oosting SF, van der Veldt AAM, GeurtsvanKessel CH, Fehrmann RSN, van Binnendijk RS, Dingemans AC, et al. mRNA-1273 COVID-19 vaccination in patients receiving chemotherapy, immunotherapy, or chemoimmunotherapy for solid tumours: a prospective, multicentre, non-inferiority trial. Lancet Oncol. 2021;22(12):1681–91. 34767759 10.1016/S1470-2045(21)00574-X PMC8577843

[55] Hippisley-Cox J, Coupland CA, Mehta N, Keogh RH, Diaz-Ordaz K, Khunti K, et al. Risk prediction of covid-19 related death and hospital admission in adults after covid-19 vaccination: National prospective cohort study. BMJ. 2021;374:n2244. 34535466 10.1136/bmj.n2244 PMC8446717

[56] Valanparambil R, Carlisle J, Linderman S, Akthar A, Millett RL, Lai L et al. Antibody response to SARS-CoV-2 mRNA vaccine in lung cancer patients: reactivity to vaccine antigen and variants of concern. MedRxiv. 2022 Jan 23:2022.01.03.22268599.

